# Cardiovascular Disease Risk Among the Mexican American Population in the Texas-Mexico Border Region, by Age and Length of Residence in United States

**DOI:** 10.5888/pcd11.130253

**Published:** 2014-04-10

**Authors:** Jennifer J. Salinas, Bassent Abdelbary, Anne Rentfro, Susan Fisher-Hoch, Joseph B. McCormick

**Affiliations:** Author Affiliations: Bassent Abdelbary, Susan Fisher-Hoch, Joseph B. McCormick, University of Texas Health Science Center, School of Public Health, Brownsville Campus, Brownsville, Texas; Anne Rentfro, University of Texas at Brownsville, Brownsville, Texas.

## Abstract

**Introduction:**

Although the relationship between health behaviors and outcomes such as smoking and obesity with longer residence in the United States among Mexican American immigrants is established, the relationship between length of residency in the United States and risk for cardiovascular disease (CVD) is not fully understood. The objective of this study was to determine the relationship between immigrant status, length of residence in the United States, age, and CVD markers in a sample of Mexican American adults living in Brownsville, Texas.

**Methods:**

We categorized participants in the Cameron County Hispanic Cohort study as immigrants in the United States for 10 years or less, immigrants in the United States for more than 10 years, or born in the United States. We conducted logistic and ordinary least squares regression for self-reported chronic conditions and CVD biomarkers.

**Results:**

We found bivariate differences in the prevalence of self-reported conditions and 1 CVD biomarker (low-density lipoprotein cholesterol) by length of residence in the middle (41–64 y) and younger (18–40 y) age groups. After adjusting for covariates, the following varied significantly by immigrant status: stroke and high cholesterol (self-reported conditions) and diastolic blood pressure, systolic blood pressure, total cholesterol, and low-density lipoprotein cholesterol (CVD biomarkers).

**Conclusion:**

The association between immigrant status, length of residence in the United States, and CVD markers varied. The effect of length of residence in the United States or immigrant status may depend on age and may be most influential in middle or older age.

## Introduction

Hispanic immigrants often have equal or better health and mortality outcomes than non-Hispanic whites, despite living in greater social and economic adversity, a trend that has been referred to as the Hispanic paradox (1). However, evidence is mounting that this paradox may pertain only to the Hispanic immigrant population. Hispanic immigrants have lower rates of cardiovascular disease (CVD) and all-cause mortality than other racial/ethnic groups in the United States (2), and some research suggests that the healthier cardiovascular profile observed among Hispanic immigrants may not be shared with US-born Hispanics (3). Hispanic immigrants have lower levels of cholesterol, triglycerides, and certain stress hormones (4,5). Moreover, evidence suggests that US-born Hispanics have CVD profiles more similar to what is expected on the basis of their low socioeconomic status (6). The understanding of nativity, socioeconomic status, and CVD risk in one Hispanic population, the Mexican American population, is still developing.

Immigrants are generally healthier and outlive their US-born counterparts, regardless of country of origin (7,8). Through the process of acculturation, however, with longer residence in the United States, immigrants adopt certain health behaviors that increase their risk for CVD, diabetes, and obesity (9–12). It is generally accepted that longer residence in the United States is associated with a linear increase in disease prevalence among immigrants and that the prevalence of disease among immigrants becomes more similar to the prevalence among the US-born over time (7). The increase in disease prevalence among immigrants results mainly from the adoption of negative health behaviors, such as drinking and smoking; these behaviors have increased among Mexican American immigrants with longer residence in the United States (13).

One possible explanation for the unusual health profile of Mexican Americans (both immigrants and US-born) is that immigrants are healthier than their US-born counterparts in general (14) and that 33.9% of Mexican Americans in the United States are immigrants (15). One study used data from the National Health and Nutrition Examination Survey to investigate the Hispanic paradox by comparing biological risk factors for CVD (ie, blood pressure, cholesterol, hemoglobin A1c [HbA1c], and inflammation) in non-Hispanic whites and Mexican Americans (4). After adjusting for socioeconomic characteristics, the study showed that US-born Mexican Americans had overall worse biological risk factor profiles for CVD (eg, higher average blood pressure, higher average levels of cholesterol, HbA1c, and inflammation) than non-Hispanic whites, but immigrant Mexican Americans had similar risk profiles to non-Hispanic whites (3). Differences in biomarkers between US-born and immigrant Mexican Americans in this study could not be explained by differences in health care access or health behaviors, suggesting that the differences may be due to a healthy immigrant effect.

Time is an important factor in the development of CVD because CVD is usually an accumulation of years of exposure to poor health habits that adversely affect biological processes. Although studies have established the relationship between health behaviors and outcomes such as smoking and obesity with longer residence in the United States among Mexican American immigrants (16), the relationship between length of residency in the United States and risk for CVD among this population is not fully understood. The objective of this study was to determine whether immigrant status and length of residence in the United States is associated with a higher prevalence of self-reported chronic conditions and worse CVD biomarker profiles in a sample of Mexican American adults living in the Texas–Mexico border region. We hypothesized that longer residence in and being born in the United States would be associated with worse CVD risk factor profiles and higher prevalence of self-reported chronic conditions than short-term residence. Because US-born Hispanics have a longer length of residence in the United States and longer potential exposure to poor health behaviors than their immigrant counterparts, we expected them to have worse CVD biological risk factor profiles and higher prevalence of self-reported chronic conditions. In addition, because disease risk increases during the life course, regardless of country of origin, we expected the effect of longer length of residence in the United States to depend on age group, or life-course stage. Because US-born Hispanics and long-term immigrants (ie, residents for more than 10 years) are more likely to engage in behaviors that increase risk for CVD, we hypothesized that we would find worse risk profiles and higher prevalence of chronic conditions among immigrants who arrived in the United States during young adulthood than among those who arrived in the United States during old age. 

## Methods

To test our hypotheses, we analyzed biological and self-reported data from the Cameron County Hispanic Cohort (CCHC), a sample of Mexican American adults living in Brownsville, Texas, a city in the Texas–Mexico border region. This study used secondary data from a study previously approved by an institutional review board. The institutional review board at the University of Texas Health Science Center at Houston certified that the analysis for this study was exempt from board review.

### Study population and sample

Brownsville provides a unique environment to test the effect of length of residence in the United States because of its proximity to Mexico and its demographic composition. Brownsville is in the Rio Grande Valley, which includes the 4 counties in the southernmost tip of Texas bordering Mexico, and approximately 31.9% of its population is immigrant. The Texas–Mexico border region has the highest proportion of Mexican Americans in the United States (17). The Mexican culture is dominant; 72.6% speak Spanish in the home (18). The Texas–Mexico border region is one of the poorest areas in the country; an estimated 30.6% of families (compared with 9.6% of families in the United States overall) live below federal poverty guidelines (19). Because the population is largely poor and of Mexican origin, it provides a natural control for the potential confounder of acculturation and socioeconomic status and allows us to focus solely on length of residence in the United States.

The CCHC is a sample of community-residing, Mexican American adults aged 18 or older (20). The sample was chosen by using a multistage cluster design from randomly selected first and third socioeconomic quartile census tracts in Brownsville (20).The total sample size used for our analysis was 1,936 (1,302 women and 634 men). Details on study recruitment and data collection are available elsewhere (20).

### Measurement

The study participants were categorized by the length of residence in the United States and immigrant status: immigrants with residence of 10 years or less (short-term immigrants), immigrants with residence of more than 10 years (long-term immigrants), and US-born. Age was categorized into 3 groups: 18–40 (young adult), 41–64 (middle age), and 65–80 (older age).We used these cut points to approximate life-course stages and correspond with periods in which CVD is most likely to proliferate. Education level was assessed as having completed high school or not having completed high school. Annual household income was categorized into 4 groups: <$10,000, $10,000 to $19,999, $20,000 or more, and missing data; we combined multiple categories of income into the single category of $20,000 or more because of low frequencies in the higher-income categories. The distribution of low income in this sample reflects the overall low socioeconomic status of Brownsville.

Chronic diseases and CVD risk factors were measured in 2 ways: by self-report of an event or diagnosis and biological measurement. An event or diagnosis of a chronic condition was measured as self-reported history of a heart attack, stroke, high cholesterol, or high blood pressure. Biological measures were diastolic blood pressure, systolic blood pressure, total cholesterol, triglycerides, high-density lipoprotein (HDL) cholesterol, and low-density lipoprotein (LDL) cholesterol. The biological markers were continuous variables for all analyses.

### Analysis

Descriptive statistics were first generated by using Stata 11 SE (21). We tabulated results by age group and length of residence in the United States versus US-born. We calculated frequencies and proportions for self-reported high blood pressure, high cholesterol, previous heart attack, and previous stroke; we calculated mean and standard deviation for each CVD biomarker. For significance testing, we used the χ^2^ test for categorical outcomes and analysis of variance for continuous outcomes; we used short-term residence as the referent category in all analyses. To test the adjusted relationship between self-reported conditions, CVD biomarkers, age group, and length of residence in the United States versus US-born, we used logistic regression for categorical outcomes and ordinary least squares regression for continuous variables. Data for each CVD biomarker were normally distributed. All models were adjusted for education, income, and sex. The analysis included sample weights. Significance was determined by an α level of .05 or less. 

## Results

We found no significant differences in sex or age by immigrant status in any age group (Table 1). In all age groups, we found significant differences by immigrant status for high school completion; in all categories, US-born participants had the highest proportion of high school completion. Although the proportion of high school completion was greatest among young adults, regardless of nativity status, the difference was significant only between the US-born and short-term immigrants. We found significant differences by immigrant status and by income for all age groups. Short-term immigrants in all age groups tended to make less than $10,000 per year; however, the proportion making less than $10,000 per year was greatest for short-term immigrants in the older age group.

**Table 1:** Demographic Characteristics, by Age Group^a^ and Years in the United States, Cameron County Hispanic Cohort, Texas^b^

Characteristic	Young Adult			Middle Age			Older Age		
	≤10 y (n = 283)	>10 y (n = 195)	US-Born (n = 346)	≤10 y (n = 164)	>10 y (n = 535)	US-Born (n = 240)	≤10 y (n = 23)	>10 y (n = 94)	US-Born (n = 56)
**Female, n (%)**	192 (59)	154 (65)	210 (47)	104 (55)	383 (61)	145 (45)	12 (42)	67 (65)	35 (46)
**Male, n (%)**	91 (41)	41 (35)	136 (53)	60 (45)	152 (39)	95 (55)	11 (58)	27 (35)	21 (54)
**Age, mean (SD), y**	30.6 (0.4)	32.6 (0.4)	28.9 (0.3)	49.2 (0.5)	52.3 (0.3)	52.3 (0.4)	70.5 (0.8)	70.7 (0.4)	72.2 (0.6)
**Completed high school, n (%)**	108 (44)	88 (56)	237 (74)^c^	46 (29)	150 (28)	147 (66)^c^	2 (9)	11 (12)	24 (50)^d^
**Income, n (%), $**									
<10,000	103 (34)	51 (21)^c^	53 (10)^c^	40 (25)	159 (29)^c^	44 (16)^c^	10 (45)	61 (61)^d^	15 (29)^d^
10,000–19,999	65 (23)	40 (14)^c^	71 (19)^c^	40 (22)	170 (32)^c^	62 (31)^c^	2 (8)	17 (20)^d^	19 (34)^d^
≥20,000	29 (8)	39 (14)^c^	93 (26)^c^	18 (15)	92 (17)^c^	76 (33)^c^	1 (5)	1 (0.3)^d^	10 (11)^d^
Missing	86 (35)	65 (51)^c^	129 (44)^c^	66 (38)	114 (22)^c^	58 (20)^c^	10 (42)	15 (19)^d^	12 (25)^d^

a Age was categorized into 3 groups: 18–40 (young adult), 41–64 (middle age), and 65–80 (older age).

b Percentages were weighted.

c *P* < .001. Referent category for analysis of variance or χ^2^ test is immigrants in United States for ≤10 years in same age group.

d *P* < .01. Referent category for statistical test is immigrants in United States for ≤10 years in same age group.

The overall prevalence of self-reported conditions increased by age group (Table 2). In the middle-age group, long-term immigrants had a significantly higher prevalence of self-reported stroke, high blood pressure, and high cholesterol than short-term immigrants, and the US born had a significantly higher prevalence of self-reported stroke than immigrants. In the young adult group, long-term immigrants had significantly higher average levels of LDL cholesterol (105.9 mg/dL vs 105.7 mg/dL) than short-term immigrants. In the older age group, the prevalence of self-reported high blood pressure was lower among long-term immigrants than among short-term immigrants. No other biomarker was significantly different by immigrant status stratified by age group.

**Table 2:** Cardiovascular Disease Markers, by Age Group^a^ and Years in the United States, Cameron County Hispanic Cohort, Texas **b**

Marker	Young Adult			Middle Age			Older Age		
	≤10 y (n = 268)	>10 y (n = 180)	US-Born (n = 337)	≤10 y (n = 164)	>10 y (n = 535)	US-Born (n = 240)	≤10 y (n = 23)	>10 y (n = 94)	US-Born ( n = 56)
**Self-reported, n (%)**									
Heart attack	1 (0.003)	0	0	1 (0.004)	10 (0.02)	12 (0.04)^c^	2 (0.15)	11 (0.14)	4 (0.10)
Stroke	1 (0.001)	0	2 (0.002)	0	16 (0.02)^c^	10 (0.04)	1 (0.05)	7 (0.06)	3 (0.04)
High blood pressure	13 (0.05)	11 (0.04)	31 (0.07)	39 (0.22)	173 (0.32)^d^	92 (0.32)	19 (0.78)	58 (0.60)^c^	35 (0.48)
High cholesterol	17 (0.08)	22 (0.12)	37 (0.11)	42 (0.21)	189 (0.37)^e^	74 (0.25)	8 (0.33)	40 (0.46)	33 (0.43)
**Measured, mean (SD)**									
Diastolic blood pressure, mm Hg	71.0 (9.4)	69.2 (9.4)	71.1 (9.7)	75.1 (9.9)	74.3 (10.2)	73.5 (9.9)	68.8 (9.4)	66.6 (9.7)	69.5 (10.7)
Systolic blood pressure, mm Hg	109.0 (11.6)	108.9 (12.1)	111.0 (12.0)	121.4 (19.5)	123.3 (17.6)	122.4 (17.1)	124.7 (16.9)	127.6 (18.9)	133.3 (17.1)
Total cholesterol, mg/dL	179.0 (40.9)	173.6 (39.7)	174.6 (40.4)	196.6 (41.0)	199.8 (42.0)	201.7 (43.3)	175.6 (43.4)	189.1 (51.1)	181.2 (39.9)
Triglycerides, mg/dL	151.4 (107.0)	139.8 (91.8)	149.1 (222.7)	179.0 (121.2)	197.5 (214.8)	239.2 (231.1)	157.4 (85.7)	196.1 (126.8)	171.6 (110.3)
High-density lipoprotein cholesterol, mg/dL	45.8 (10.5)	48.2 (11.3)	44.6 (10.8)	46.6 (11.9)	47.8 (12.1)	47.8 (13.1)	47.8 (11.6)	48.1 (12.6)	48.0 (12.9)
Low-density lipoprotein cholesterol, mg/dL	105.7 (34.3)	105.9 (38.3)^c^	103.3 (30.9)	117.0 (33.0)	117.8 (34.1)	114.5 (32.5)	96.4 (34.6)	101.9 (35.3)	100.4 (35.8)

a Age was categorized into 3 groups: 18–40 (young adult), 41–64 (middle age), and 65–80 (older age).

b Not all survey participants answered all questions, so *n*’s in this table may vary from *n*’s in Table 1. In addition, percentages were calculated according to the number of participants who answered the question.

c *P* < .001. Referent category for analysis of variance or χ^2^ test is immigrants in United States for ≤10 years in same age group.

d *P* < .01. Referent category for statistical test is immigrants in United States for ≤10 years in same age group.

e *P* < .05. Referent category for statistical test is immigrants in United States for ≤10 years in same age group.

Being US-born was significantly associated with having had a stroke (OR = 4.3, *P* = .04) (Table 3). Long-term immigrants were significantly more likely than short-term immigrants to report having high cholesterol (OR = 1.9, *P* = .004). Long-term immigrants had on average lower diastolic blood pressure (β = −1.40, *P* = .04) than short-term immigrants. The US-born had on average significantly higher systolic blood pressure (β = 3.45, *P* = .003) but lower average levels of total cholesterol (β = −4.19, *P* = .03) than short-term immigrants. Finally, US-born had on average lower levels of LDL cholesterol (β = −3.77, *P* = .03) than short-term immigrants.

**Table 3:** Regression Analysis of Cardiovascular Disease Markers, by Age Group^a^ and Years in the United States, Cameron County Hispanic Cohort, Texas

Marker	Nativity^b^		Age Group^c^	
	>10 Years in United States	US Born	Middle Age	Old Age
**Self-reported condition^c^, OR (95% CI)**				
Heart attack	1.2 (0.3–4.5)	1.4 (0.4–5.0)	15.0 (3.9–58.6)	84.1 (26.5–267.4)
Stroke	3.0 (0.5–20.1)	4.3 (1.1–17.6)	6.8 (2.2–20.7)	11.6 (3.5–38.0)
High blood pressure	1.2 (1.0–1.6)	1.5 (1.0–2.3)	6.6 (4.1–10.6)	18.9 (10.1–32.5)
High cholesterol	1.9 (1.2–2.8)	1.4 (0.4–2.5)	3.1 (1.9–4.6)	5.0 (2.5–10.6)
**Measured, β (SE)**				
Diastolic blood pressure quartiles	−1.40 (0.64)	−0.535 (0.57)	3.98 (0.49)	−2.12 (0.80)
Systolic blood pressure quartiles	1.08 (1.1)	3.45 (1.0)	13.0 (0.85)	18.6 (2.1)
Total cholesterol	−0.342 (4.5)	−4.19 (1.7)	23.3 (4.0)	11.8 (6.7)
Triglycerides	2.37 (10.3)	14.4 (13.7)	60.5 (15.6)	39.1 (19.4)
High-density lipoprotein cholesterol	1.33 (0.71)	−0.829 (0.77)	1.54 (0.57)	2.57 (1.3)
Low-density lipoprotein cholesterol	0.844 (3.6)	−3.77 (1.6)	11.2 (2.2)	−2.77 (5.9)

Abbreviations: OR, odds ratio; CI, confidence interval; SE, standard error.

a Age was categorized into 3 groups: 18–40 (young adult), 41–64 (middle age), and 65–80 (older age).

b Referent category is immigrants in United States for ≤10 years.

c Referent category is young adult.

## Discussion

We found bivariate differences in the prevalence of self-reported conditions and 1 CVD marker (LDL cholesterol) by length of residence in the United States in middle and younger ages. After adjusting for covariates, we found significant differences by immigration status for the following self-reported conditions and CVD biomarkers: stroke and high cholesterol (self-reported) and diastolic blood pressure, systolic blood pressure, total cholesterol, and LDL cholesterol (CVD biomarkers). 

Although some researchers have found significant differences in CVD markers between Mexican American immigrants and US-born Mexican Americans (5,22), our study demonstrates that the differences between immigrant and US-born Mexican Americans are primarily differences between short-term immigrants and the US-born. Although behavioral changes may take place in the United States during younger ages, they may not translate into disease until middle or older age. Other mechanisms, such as genetics or regional variations, may be responsible for the differences in CVD rates found in other studies (10). For example, one mechanism may be the higher prevalence of diabetes along the Texas–Mexico border (17,23), and some evidence suggests that ancestry and certain genetic markers may be linked to subclinical CVD (24).

Socioeconomic status may mediate the relationship between length of residence in the United States and health behaviors and mental health. For example, among older Mexican Americans, longer length of residence in the United States is associated with better cognitive status and physical functioning, due in part to improvements in economic status after arrival in the United States (25). A study investigating differences in obesity by education level found a gradient effect of length of residence in the United States only among immigrants with low levels of education (26). Because some studies have found a relationship between CVD and socioeconomic factors, the lack of a difference between short-term and long-term immigrants in our study could also be related to low educational levels, low rates of insurance coverage, or low income overall in our sample.

Despite the increase in risk for most diseases with age, few studies, if any, have examined age-specific trends of nativity status in terms of both self-reported CVD events and diagnoses and CVD biomarkers. A study on older Mexican Americans found a relationship between length of residence in the United States and risk for hypertension and other related self-reported conditions. For example, one study (27) found an immigrant advantage for older Mexican American men in the Hispanic Established Populations for the Epidemiologic Studies of the Elderly (Hispanic EPESE). Greater use of the English language was associated with a higher prevalence of hypertension in Mexican American men, smoking in Mexican American women, and all-cause mortality in both sexes in the Hispanic EPESE (28). Although the findings in our study on the nativity effects on the prevalence of disease in older age are consistent with the findings of previous studies, no study has examined this relationship across the life course, as has been done here.

The lack of a significant difference between immigrant groups and the US-born in the young adult group may be due to the growing rates of obesity, diabetes, and CVD in Mexico (11). One study found a higher prevalence of hypertension in a national sample of Mexicans than in a sample of Mexican Americans (29). It is likely that younger immigrants from Mexico are less healthy than older generations relative to their US-born counterparts. Future studies comparing CVD markers in older and younger age groups in the Mexican population should be considered to evaluate changing health status by age group in Mexico.

This study has several limitations, most notably, the lack of longitudinal data to track progression of CVD in this population, particularly among the young adult group. It would be important to know whether differences in CVD arise as these age groups continue along the life course. Another potential limitation is the location of this study in the Texas–Mexico border region, where the population is predominantly poor and Mexican American. It may be that Mexican American immigrants who live in more advantaged and heterogeneous areas of the United States may experience CVD differently at younger ages compared with US-born Mexican Americans. Additionally, because this study was a cross-sectional investigation, the underlying assumption was that that all immigrant cohorts were the same. This assumption may have created bias — immigrant streams and catalysts to immigration depend on time and may change. The preferred method for studying the relationship between immigration and health outcomes would be over time in a longitudinal study. Future studies using a longitudinal approach could identify changes in behavior, socioeconomic status, and health conditions with length of residence in the United States.

Despite its limitations, this study provides new insight into the proliferation of CVD among Mexican Americans, suggesting that life course and length of time in the United States may influence differences by nativity status in this population. Adaptation to a host country changes as immigrants age. Understanding how these 2 processes influence each other will provide much-needed information about the Hispanic paradox and Hispanic health overall. This understanding is important as Hispanics become the largest racial/ethnic group in the country and because Mexican Americans are the majority within this racial/ethnic group. The policy and economic implications for understanding these processes are far reaching.
